# Supplementary material for: Conidium Specific Polysaccharides in Aspergillus fumigatus
Source: J Fungi (Basel). 2023 Jan 24;9(2):155. doi: 10.3390/jof9020155 (PMC9964227; doi:10.3390/jof9020155)
Supplement: Supplementary file 1 [file jof-09-00155-s001.zip › jof-2130314-supplementary file.pdf]

Table S1. *A. fumigatus* strains used in this study.

Strains	Deleted genes	references
KU80ΔpyrG	Parental strain	[13]
Δ*ugm1*	AFUA_4G12690	[30]
Δ*och1-4* (GT32 family)	AFUA_8G02040<br>AFUA_6G14040<br>AFUA_1G01380<br>AFUA_5G08580	[31]
ΔGT62	MNN9 AFUA_2G01450<br>ANP1 AFUA_4G06870<br>VAN1 AFUA_2G15910	[12]
Δ*gel1-2* (GH-72)	GEL1 AFUA_2G01170<br>GEL2 AFUA_6G11390	[51]
ΔExoG (sextuple GH-55)	EXG5 AFUA_6G11980<br>EXG6 AFUA_6G13270<br>EXG7 AFUA_3G07520<br>EXG8 AFUA_1G14450<br>EXG9 AFUA_2G00430<br>EXG10 AFUA_4G03350	[48]
ΔEndoG	ENG1 AFUA_1G04260 (GH81)<br>ENG2 AFUA_2G14360 (GH16)<br>ENG3 AFUA_1G05290 (GH16)<br>ENG4 AFUA_5G02280 (GH16)<br>ENG5 AFUA_4G13360 (GH16)	[39]
ΔEndoG/ΔExoG	EXG5 AFUA_6G11980<br>EXG6 AFUA_6G13270<br>EXG7 AFUA_3G07520<br>EXG8 AFUA_1G14450<br>EXG9 AFUA_2G00430<br>EXG10 AFUA_4G03350<br>ENG1 AFUA_1G04260<br>ENG2 AFUA_2G14360<br>ENG3 AFUA_1G05290<br>ENG4 AFUA_5G02280<br>ENG5 AFUA_4G13360	[48]
Δ5-GH17	BGT1 AFUA_1G11460<br>BGT2 AFUA_3G00270<br>BGT3 AFUA_5G08780<br>SCW4 AFUA_6G12380<br>SCW11 AFUA_8G05610	[47]

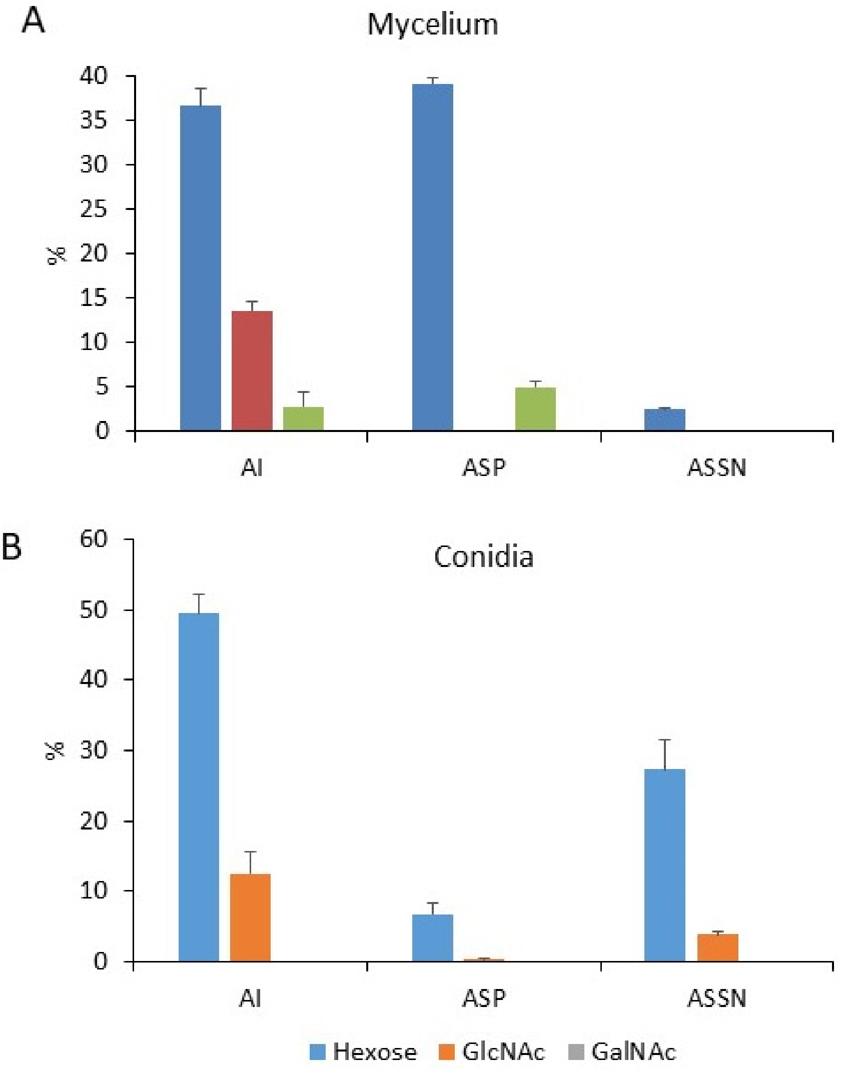

Figure S1. **Sugar analysis of cell wall fractions of mycelium and conidia from *Δku80 A. fumigatus* parental strain, grown on solid malt medium.** A, composition of mycelium cell wall; B, composition of conidia cell wall. The composition is expressed in % of total cell wall sugar. Analyses were performed on three independent biological replicates.

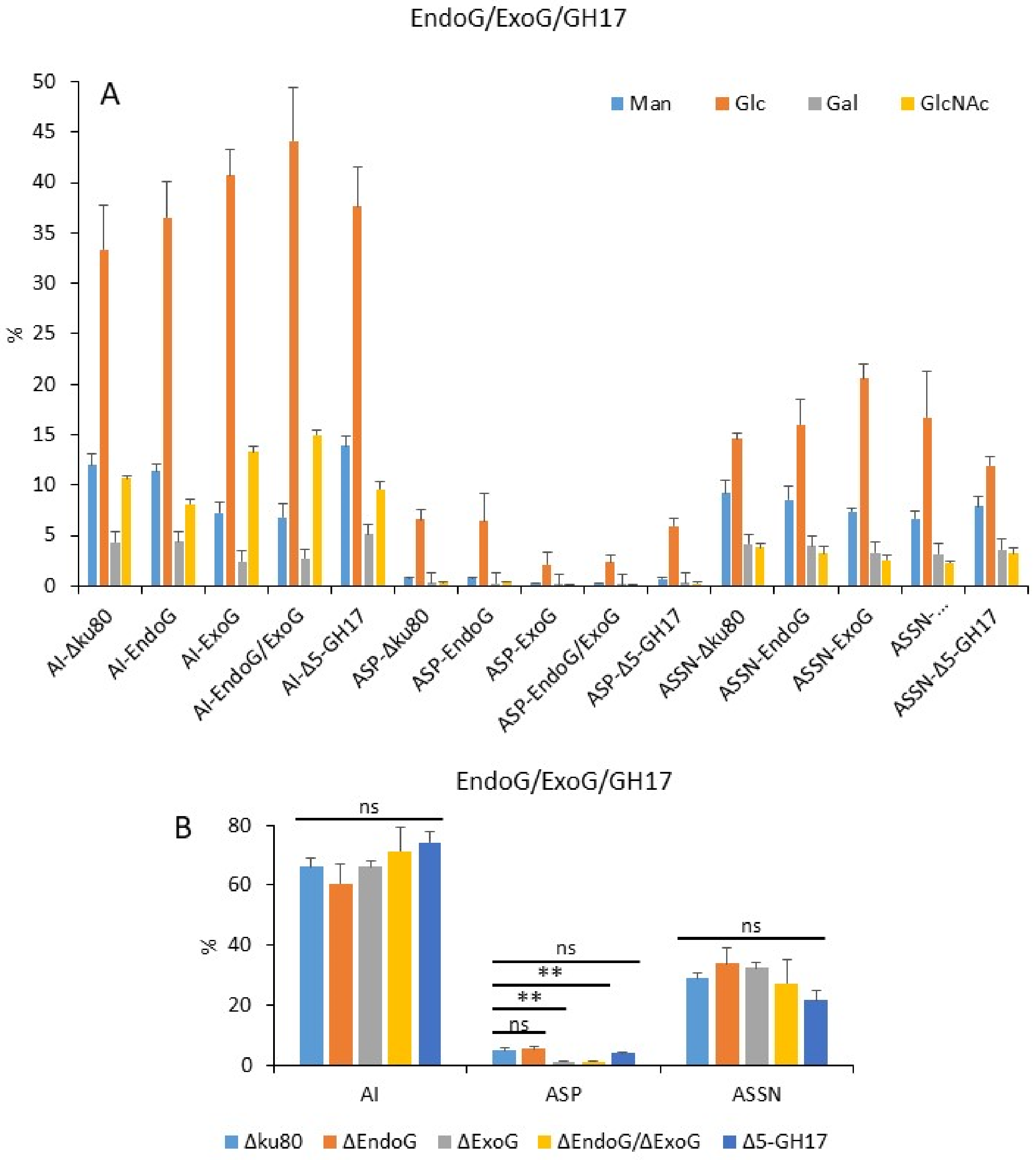

Figure S2. **Sugar analysis of cell wall fractions of conidia from *Δku80* (parental strain), endo-β-glucanase, exo-β-glucanase and transglycosylase deficient mutants.** ΔendoG mutant is a quintuple mutant deficient in GH16 and GH81 family members, ΔexoG is a sextuple mutant deficient in GH55 family members, endoG/exoG is an undecuple mutant deficient in GH16, GH55 and GH81 family members. Δ5-GH17 is a quintuple mutant deficient in GH17 family members (see Table S1). (**A**), monosaccharide composition; (**B**), β-glucan composition. The composition is expressed in % of total cell wall sugar in panel A and in % of total β-glucan in panel B. The experiments were performed on three independent biological replicates.

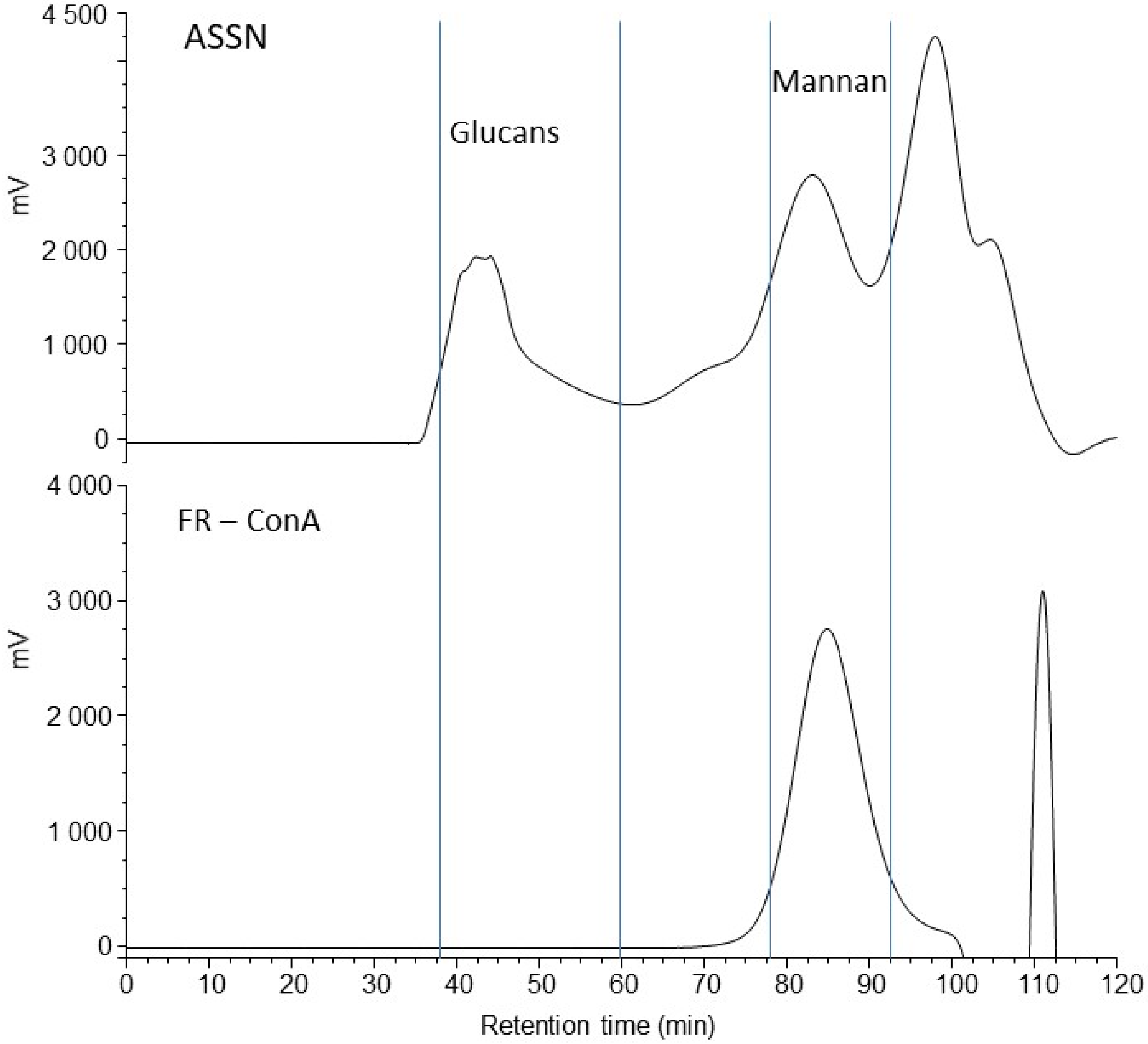

Figure S3. Gel filtration chromatography on Superdex S200 of total ASSN fraction from the ugm1 mutant (ASSN) and the corresponding Concanavalin-A bound fraction (FR-ConA). None of the fractions was submitted to laminarinase digestion.

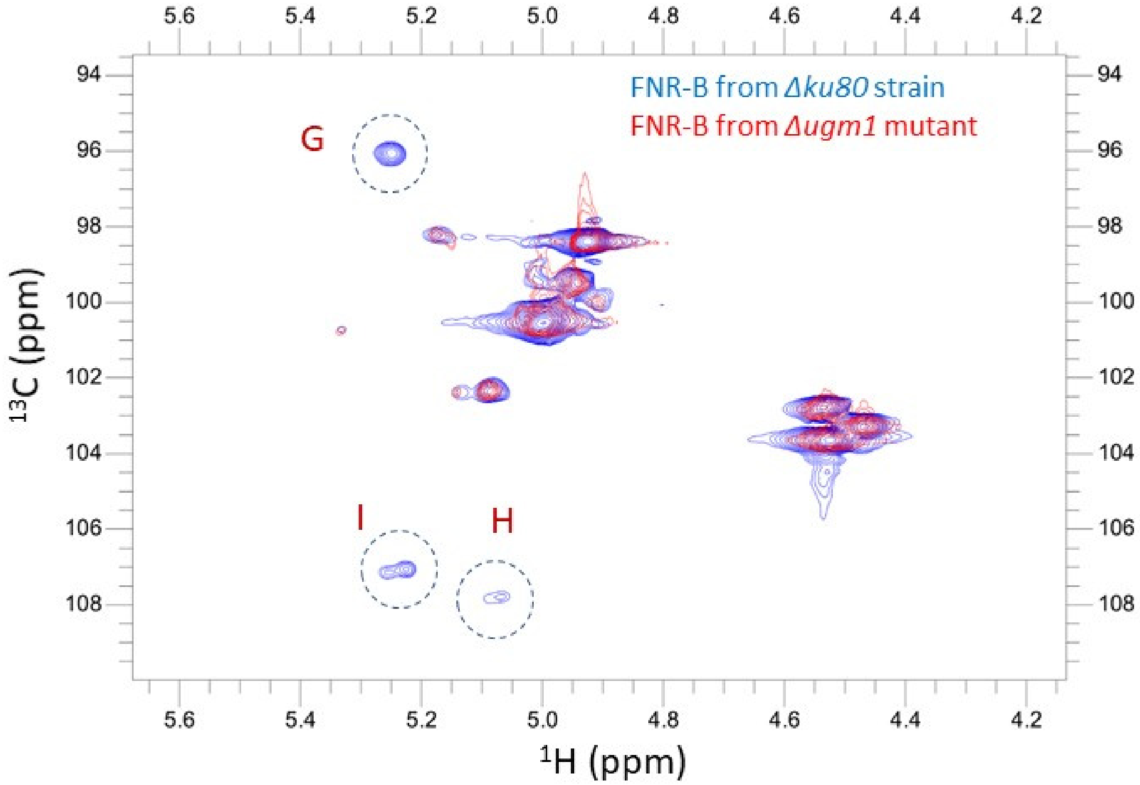

Figure S4. Comparison of the $^{1}H^{13}C$-HSQC anomeric region of FNR-B fractions extracted from the *Δku80* parental strain (blue) and the *Δugm1* mutant (red). The mannan extracted from the *Δugm1* mutant lacks galactofuranose residues H and I (5-5.3 ppm ^{1}H; 107-108 ppm ^{13}C) and an unidentified residue (G, 5.25 ppm ^{1}H ; 96.1 ppm ^{13}C) indicated by dashed blue circles. **Quantification of galactofuranose based on the integration of anomeric signals: 2.5% of total galactose residues and no covalent linkage with G3Man has been identified.**

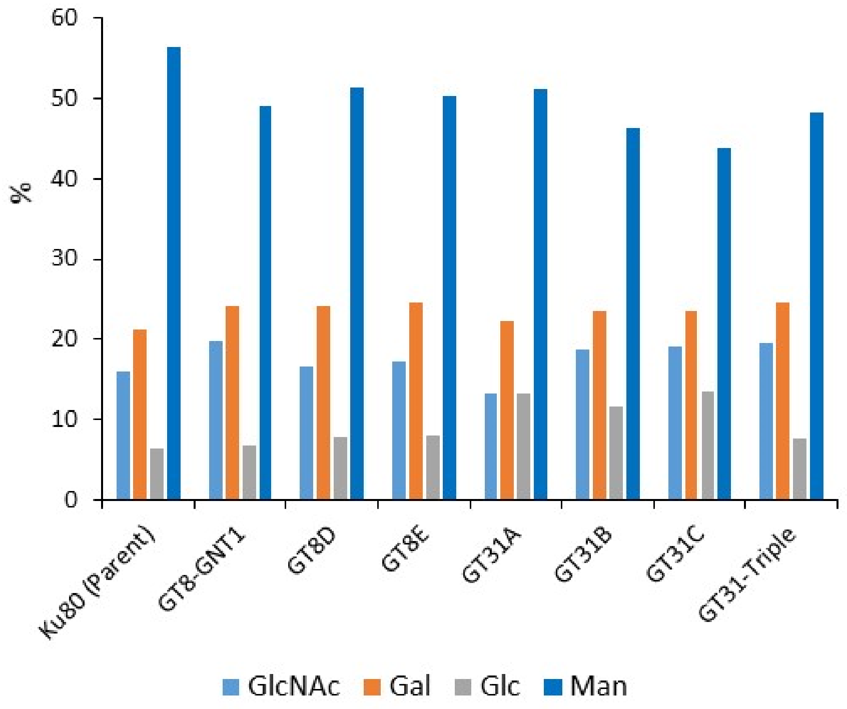

Figure S5. Monosaccharide composition of the FNR fractions isolated from *A. fumigatus* glycosyltransferase mutants conidia ASSN. Single mutants: GT8-GNT1, AFUA_8G02690; GT8D: AFUA_8G01730; GT8E: AFUA_5G09070; GT31A: AFUA_2G17320; GT31B: AFUA_6G00520; GT31C: AFUA_3G07220; GT31-Triple: triple mutant Δgt31A/Δgt31B/Δgt31C.
